# Skiagraphs in the Diagnosis of Thoracic Disease

**Published:** 1897-04

**Authors:** 


### SKIAGRAPHS IN THE DIAGNOSIS OF THORACIC DISEASE.

At the December meeting of the Paris Academy of Sciences, as reported in the *Medical Week*, Prof. Ch. Bouchard gives some interesting facts of the use of the X-ray as a factor in the diagnosis of thoracic disease. On several occasions radioscopy revealed morbid conditions which were not perceived on physical examination, even when repeated with special reference the readiosopic indications.

On examining by the aid of the Rœntgen rays a suspected thorax, I discovered toward one apex a somewhat dark spot in which the dark tint was unevenly distributed. Applying the ear to the area thus brought to my attention, I detected the existence of friction sounds.

A patient was presented to me without any information as to his condition, and without my being able to see him in the darkened room. The image of one side of the thorax was light, whereas on the left side it was dark throughout. I therefore arrived at the conclusion that there was either a pleural effusion or tubercular infiltration of the entire left lung. Having noticed that the mediastinum was not deviated toward the right I was enabled to exclude pleurisy. Other means of exploration fully confirmed my diagnosis of tubercular infiltration of the left lung.

I have also distinctly shown a tumor, formed on the right side of the vertebral column in a little girl, by a mass of enlarged tracheo-bronchial glands.

A patient who had just been admitted into hospital and had only been subjected to a cursory examination, was brought to me because he presented pulsation on the right side of the sternum, and it was desired to ascertain the result obtained by radioscopy in case he suffered from aneurism of the aorta. In the thorax on the right side I found an abnormal dark area, continuous with the shadow of the sternum; but I also noted that there was no shadow of the heart on the left side. I therefore concluded that this was a case not of aneurism of the aorta, but of ectopy of the heart, which necessarily must beat on the right side, inasmuch as it was not situated on the left. This diagnosis was immediately confirmed by palpation, percussion, and auscultation.

In the case of another patient, in whom by the usual means I had diagnosed aneurism of the aortic arch, the phosphorescent screen showed the situation, shape, and size of the tumor.

Apart from aneurisms, I have also made out dilatation of the aorta on the right and left side of the sternum from the presence of a curve of a slightly darker tint than the rest of the thorax, representing the distended portions of the vessel.

Likewise I have recognized compensatory hypertrophy of the heart in arterial or renal schlerosis by the fact that, in this case the auricles were seen beating to the right of the sternum.

These facts, added to those to which I have already called attention, show that the Rœntgen rays, from the use of which surgeons derive such great benefit in investigation of osseous lesions, and in the search for foreign metallic bodies, may be employed by physicians with equal advantage, at any rate as far as diseases of the thorax are concerned.

The results so far obtained from the application of radioscopy to the investigation of diseases of the abdomen have been less satisfactory. I have clearly distinguished the arch formed by the convex surface of the liver; but I have rarely been able to make out the lower limit of this organ. I have seen nothing of the kidneys, nor have I been able to distinguish a large cancer of the stomach. Only once have I, dimly and indistinctly, seen the head of the fetus in a case of pregnancy. The stomach, however, appears distinctly as a light spot on the dark background of the abdomen, providing it be distended by gases.

By using powerful coils, by which the tension of the alternating currents can be raised so as to furnish sparks of 40 centimeters, I have succeeded in overcoming the resistence of the thorax in adults to the Rœntgen rays, so as to show on the phosphorescent screen the details of the thoracic organs. By still further increasing the power of the penetration of the rays, I hope to be able to make them pass also through the far more resistent abdominal organs.—*N. A. Practitioner.*

